# Supplementary material for: Echocardiography and MALDI-TOF Identification of Myosin-Binding Protein C3 A74T Gene Mutations Involved Healthy and Mutated Bengal Cats
Source: Animals (Basel). 2022 Jul 12;12(14):1782. doi: 10.3390/ani12141782 (PMC9312240; doi:10.3390/ani12141782)
Supplement: Supplementary file 1 [file animals-12-01782-s001.zip › animals-1778747-supplementary.pdf]

Supplementary material file 1

Availability of data and materials

The datasets generated and/or analysed during the current study are available in the NCBI database repository (myosin binding protein C3 in domestic cat; gene ID: 101094698), https://www.ncbi.nlm.nih.gov/gene/101094698.

DNA Sequencing

```
Myosin Binding Protein C3: A31P and A74T
       ....|....| ....|....| ....|....| ....|....| ....|....|
                         10         20         30         40         50
180013-BA-     CGACAGCTCT GCTGTGTTCG AGGCCGAGAC AGAGCGGTCA GGAGTAAAGG

       ....|....| ....|....| ....|....| ....|....| ....|....|
                         60         70         80         90        100
180013-BA-     TGCGCTGGCA GCGGGGGGGC AGTGACATCA GCGCCAGTGA CAAGTATGGC

       ....|....| ....|....| ....|....| ....|....| ....|....|
                        110        120        130        140        150
180013-BA-     CTAGCAGCCG AGGGCACGAG GCACACTCTG ACAGTGCGGG ACGTGGGCCC

       ....|....| ....|....| ....|....| ....|....| ....|....|
               160        170        180        190        200
180013-BA-     CGCCGACCAG GGACCCTACG CAGTCATCGC TGGCTCCTCC AAGGTCAAGT

       ....|

180013-BA-     TTGAA
```
